# Dipeptidyl Peptidase 4 Inhibitors Decrease the Risk of Hepatocellular Carcinoma in Patients With Chronic Hepatitis C Infection and Type 2 Diabetes Mellitus: A Nationwide Study in Taiwan

**DOI:** 10.3389/fpubh.2021.711723

**Published:** 2021-09-17

**Authors:** Wei-Hao Hsu, Shu-Ping Sue, Hsiu-Ling Liang, Chin-Wei Tseng, Hsiu-Chu Lin, Wei-Lun Wen, Mei-Yueh Lee

**Affiliations:** ^1^Division of Endocrinology and Metabolism, Department of Internal Medicine, Kaohsiung Medical University Hospital, Kaohsiung Medical University, Kaohsiung, Taiwan; ^2^Department of Internal Medicine, Kaohsiung Municipal Siaogang Hospital, Kaohsiung Medical University, Kaohsiung, Taiwan; ^3^Department of Nursing, Kaohsiung Medical University Hospital, Kaohsiung Medical University, Kaohsiung, Taiwan; ^4^Faculty of Medicine, College of Medicine, Kaohsiung Medical University, Kaohsiung, Taiwan

**Keywords:** dipeptidyl peptidase 4 inhibitors, diabetes mellitus, hepatitis C virus infection, hepatocellular carcinoma, antidiabetic drugs

## Abstract

**Introduction:** Dipeptidyl peptidase 4 inhibitors (DPP-4 inhibitors) are incretin-based oral antidiabetic drugs. Previous studies have shown an association between increased plasma activity of DPP-4 and chronic hepatitis C virus (HCV) infection. Dipeptidyl peptidase 4 inhibitors may be associated with preventing the development of chronic HCV infection. The aim of this study was to investigate whether the use of DPP-4 inhibitors is associated with a decreased risk of hepatocellular carcinoma (HCC) in patients with diabetes mellitus (DM) and chronic HCV infection.

**Methods:** In this retrospective cohort study, we enrolled patients with type 2 diabetes and chronic HCV infection from the National Health Insurance Research Database (NHIRD) in Taiwan. The patients were divided into two groups (DPP-4 inhibitor cohort and non-DPP-4 inhibitor cohort) according to whether or not they received DPP-4 inhibitor treatment.

**Results:** Multivariate Cox proportional hazard regression analysis showed a significantly lower risk of HCC in the patients who took DPP-4 inhibitors compared to those who did not. Kaplan-Meier survival analysis demonstrated a significantly higher HCC-free rate in the DPP-4 inhibitor cohort than in the non-DPP-4 inhibitor cohort.

**Conclusion:** The use of DPP-4 inhibitors was associated with a lower risk of HCC in patients with type 2 DM and chronic HCV infection.

## Introduction

Primary liver cancer is the third leading cause of cancer mortality worldwide, of which hepatocellular carcinoma (HCC) is the most common type (1). The major traditional risk factors for HCC are chronic hepatitis B virus (HBV) and hepatitis C virus (HCV) infection, drinking alcohol, smoking tobacco, and exposure to aflatoxins (2). In addition, recent studies have shown that diabetes mellitus (DM), non-alcoholic fatty liver disease, and obesity are also risk factors for HCC (3). Several epidemiological studies have reported an association between DM and HCC. A meta-analysis of 13 cohort and 13 case-control studies found that patients with DM had a 2.5-fold higher risk of HCC (4). In addition, a systematic review of epidemiologic investigations on DM and HCC in Japan including 19 cohort studies, one pooled-analysis of seven cohort studies, and seven case-control studies also strongly supported an increased risk of HCC among patients with DM (5). Accordingly, there is increasing interest in whether antidiabetic medications can influence the development and/or progression of HCC in patients with DM. A meta-analysis of observational studies found that metformin was associated with a decreased incidence of HCC in patients with DM (6). Moreover, in another systematic review and meta-analysis, the type and dose of antidiabetic drugs appeared to influence the risk of HCC in diabetic patients (7).

Dipeptidyl peptidase 4 (DPP-4) inhibitors are incretin-based oral antidiabetic drugs that inhibit the degradation of glucagon-like peptide-1 and glucose-dependent insulinotropic peptide, leading to stimulation of insulin secretion and inhibition of glucagon secretion (8). Exogenous sulfonylurea and insulin treatment have been associated with an increased risk of HCC in patients with HCV infection and DM (9). However, DPP-4 inhibitor treatment has not been shown to promote the development of tumors in mice (10). In addition, a previous meta-analysis found that patients with type 2 DM who were treated with DPP-4 inhibitors did not have a higher risk of developing cancer than those who received other drugs or a placebo (11). Moreover, another systematic review and meta-analysis with a mean follow-up of 1.5 years found no evidence of an association between DPP-4 inhibitors and cancer in patients with type 2 DM (12). Thus, DPP-4 inhibitors may not increase the risk of HCC in patients with DM.

Riva et al. reported an association between increased DPP-4 plasma activity and chronic HCV infection via the production of a truncated form of the chemokine CXCL10 (13). In addition, they identified an association between truncated CXCL10 and failure to achieve spontaneous clearance of acute HCV infection (13). These findings suggest that DPP-4 inhibitors may be associated with preventing the development of chronic HCV infection, and therefore DPP-4 inhibitors may be associated with the primary prevention of HCC in patients without chronic HCV infection. The aim of this study was to investigate whether the use of DPP-4 inhibitors is associated with a decreased risk of HCC in patients with DM and chronic HCV infection.

## Materials and Methods

### Data Source

This population-based retrospective cohort study used data from the National Health Insurance Research Database (NHIRD) in Taiwan. The NHIRD was established in 1995 and covers approximately 99% of the residents in Taiwan. It is one of the largest and most complete nationwide population-based datasets in Taiwan. In this study, we used the Longitudinal Health Insurance Database 2000, which is a subset of the NHIRD and includes a representative sample of one million randomly sampled patients from the year 2000 registry of all enrollees in the National Health Insurance program using a systematic sampling method. All diseases in the dataset were classified using International Classification of Diseases, Ninth Revision, Clinical Modification (ICD-9-CM) diagnostic codes.

### Study Population

Patients with a diagnosis of type 2 DM (ICD-9-CM codes: 250.00, 250.02, 250.10, 250.12, 250.20, 250.22, 250.30, 250.32, 250.40, 250.42, 250.50, 250.52, 250.60, 250.62, 250.70, 250.72, 250.80, 250.82, 250.90, and 250.92) and a new diagnosis of chronic HCV infection (ICD-9-CM codes: 070.44, and 070.54) from 2009 to 2010 were identified. The index date was defined as the date when the patients were newly diagnosed with HCV infection. The patients were followed from the index date to death, the occurrence of HCC (ICD-9-CM code: 155), or December 31, 2013, whichever occurred first. The patients with a diagnosis of HCV before 2009, a diagnosis of type 1 DM (ICD-9-CM codes: 250.01, 250.03, 250.11, 250.13, 250.21, 250.23, 250.31, 250.33, 250.41, 250.43, 250.51, 250.53, 250.61, 250.63, 250.71, 250.73, 250.81, 250.83, 250.91, and 250.93), any previous cancer diagnosis (ICD-9-CM codes: 140.x−239.x), a diagnosis of HCC before the index date, age <20 years old, or having incomplete data were excluded. The patients were divided into two groups (DPP-4 inhibitor cohort and non-DPP-4 inhibitor cohort) according to whether or not they received DPP-4 inhibitor treatment. The study protocol was approved by the Institutional Review Board of Kaohsiung Medical University Hospital (KMUHIRB-F(I)-20170014).

### Study Design

The following covariates and comorbidities were analyzed in this study: age, sex, insurance range (New Taiwan Dollar (NTD. <15,840; NTD. 15,840–25,001; NTD. >25,001), liver cirrhosis (ICD-9-CM codes: 571.2, 571.5), HBV infection (ICD-9-CM codes: 070.20–070.23, 070.30–070.33, V02.61), other antidiabetic medications (such as sulfonylureas, metformin, pioglitazone, acarbose, meglitinides, and insulin), anti-HCV agents (ribavirin and peginterferon), and duration of diabetes (months). The two groups (DPP-4 inhibitor and non-DPP-4 inhibitor cohorts) were matched at a 1:1 ratio using these comorbidities and covariates using propensity score matching. The study outcome was the occurrence of HCC in the two cohorts.

### Statistical Analysis

Pearson's chi-squared test or Fisher's exact test was used to compare each categorical variable between the DPP-4 inhibitor and non-DPP-4 inhibitor groups. The independent *t*-test was used to compare continuous variables. Intake duration (years) and cumulative defined daily dose of DPP-4 inhibitors were calculated from the reimbursement database, and tertiles of intake duration and cumulative defined daily dose were used for analysis. The incidence rate of HCC (per 1,000 person-years) was calculated for each cohort including the DPP-4 inhibitor group, the non-DPP-4 inhibitor group, each tertile of cumulative defined daily dose in the DPP-4 inhibitor group, and each tertile of intake duration in the DPP-4 inhibitor group. Crude hazard ratios and 95% confidence intervals (CIs) with the non-DPP-4 inhibitor group as the reference group for the risk of HCC were calculated for above each group except the non-DPP-4 inhibitor group. Multivariate Cox proportional hazard regression analysis was used to calculate the hazard ratios and 95% CIs for the risk of HCC by adjusting for age, sex, insurance range, comorbidities, other antidiabetic medications, anti-HCV agents and duration of diabetes. Kaplan-Meier survival curve analysis was used to assess the 5-year HCC-free rates in the DPP-4 inhibitor and non-DPP-4 inhibitor groups, and the log-rank test was used to test differences between the two groups. All statistical analyses were performed using SAS software (SAS System for Windows, v. 9.3, SAS Institute, Cary, NC, USA). A *P*-value < 0.05 was considered to indicate a statistically significant difference.

## Results

**Figure 1:**
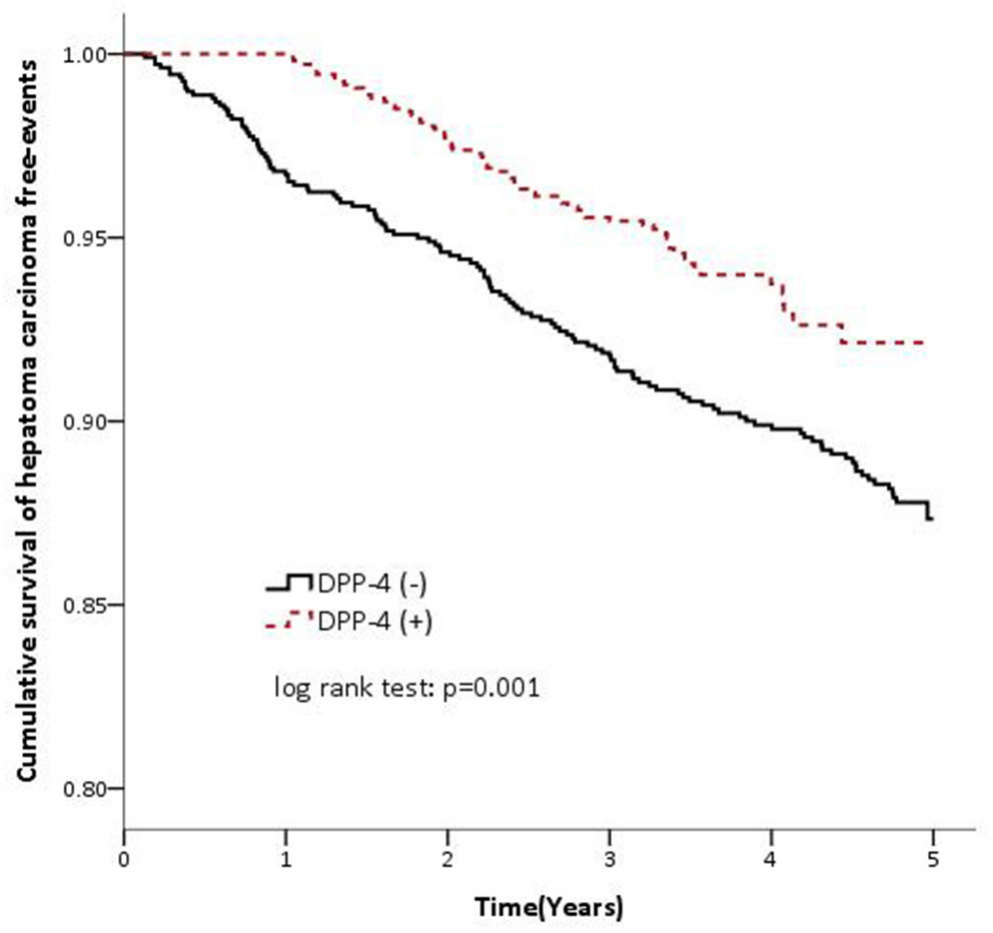
Cumulative survival of hepatoma carcinoma (hepatocellular carcinoma) free events in the DPP-4 inhibitor cohort (dashed line) and non-DPP-4 inhibitor cohort (solid line).

A total of 2,166 patients including 1,064 females and 1,102 males with type 2 DM and HCV infection were included. The demographic and medical characteristics of the DPP-4 inhibitor and non-DPP-4 inhibitor groups are shown in Table 1. The mean age was 60.41 years in the non-DPP-4 inhibitor group, and 59.49 years in the DPP-4 inhibitor group. There were no significant differences in age, insurance range, comorbidities including HBV infection and liver cirrhosis, the use of antidiabetic medications except for DPP-4 inhibitors, the use of anti-HCV agents, and the duration of diabetes between the two groups. There were 28.65 and 16.34 cases of HCC per 1,000 person-years from 2009 to 2013 in the non-DPP-4 inhibitor group and DPP-4 inhibitor group, respectively (Table 2). After adjusting for age, sex, insurance range, comorbidities, other antidiabetic medications, anti-HCV agents and duration of diabetes using multivariate Cox proportional hazard regression analysis, the results revealed that the patients who took DPP-4 inhibitors had a significantly lower risk of HCC [adjusted hazard ratio (aHR), 0.59; 95% CI, 0.43–0.79; *P* = 0.001) compared to those who did not take DPP-4 inhibitors. In addition, the patients who took DPP-4 inhibitors with a cumulative defined daily dose of more than 392 (aHR, 0.33; 95% CI, 0.18–0.58; *P* < 0.001) and those who took DPP-4 inhibitors for more than 1.49 years (aHR, 0.17; 95% CI, 0.09–0.34; *P* < 0.001) also had significantly lower risks of HCC compared to those who did not take DPP-4 inhibitors (Table 2). The Kaplan-Meier survival analysis demonstrated a significantly higher HCC-free rate in the DPP-4 inhibitor group than in the non-DPP-4 inhibitor group (log-rank test, *P* = 0.001; Figure 1).

**Table 1:** Demographic data.

	**DPP-4i(–) (***n*** = 1,083)**		**DPP-4i(+) (***n*** = 1,083)**		
	***N***	**(%)**	***N***	**(%)**	***p*-values**
Age, mean	60.41	(11.05)	59.49	(10.84)	0.052
<40	38	(3.5)	42	(3.9)	0.449
40–59	507	(48.6)	519	(47.9)	
≥60	538	(49.7)	522	(48.2)	
Gender					
Female	535	(49.4)	529	(48.8)	0.796
Male	548	(50.6)	554	(51.2)	
Insurance range					
< NTD. 15,840	501	(46.3)	494	(45.6)	0.932
NTD. 15,840–NTD. 25,001	356	(32.9)	364	(33.6)	
>NTD. 25,001	226	(20.9)	225	(20.8)	
Comorbidities					
HBV	234	(21.6)	257	(23.7)	0.238
Liver cirrhosis	858	(79.2)	857	(79.1)	0.958
Other antidiabetic medications					
Sulfonylureas	619	(57.2)	627	(57.9)	0.728
Metformin	725	(66.9)	698	(64.5)	0.222
Pioglitazone	107	(9.9)	119	(11.0)	0.399
Acarbose	191	(17.6)	192	(17.7)	0.955
Meglitinides	86	(7.9)	89	(8.2)	0.813
Insulin	59	(5.4)	74	(6.8)	0.179
Anti-HCV agents	73	(6.7)	84	(7.8)	0.362
Duration of diabetes (months)					
<36	299	(27.6)	311	(28.7)	0.566
≥36	784	(72.4)	772	(71.3)	

*DPP-4i(–), non-dipeptidyl peptidase 4 inhibitor cohort; DPP-4i(+), dipeptidyl peptidase 4 inhibitor cohort; NTD, New Taiwan Dollar; HBV, hepatitis B virus; HCV, hepatitis C virus. Age is shown as geometric mean*.

**Table 2:** The risk of hepatocellular carcinoma in patients with type 2 DM and chronic HCV infection.

	**Case no**	**Per 1,000 person year**	**Crude hazard ratio (95% CI)**	***p*-values**	**Adjusted hazard ratio (95% CI)**	***p*-values**
DPP-4i(–)	130	28.65	Reference		Reference	
DPP-4i(+)	65	16.34	0.57 (0.42–0.78)	<0.001	0.59 (0.43–0.79)	0.001
Cumulative DDD						
<153	27	21.21	0.75 (0.49–1.14)	0.171	0.78 (0.51–1.19)	0.241
153–392	25	19.73	0.70 (0.45–1.07)	0.100	0.70 (0.45–1.08)	0.111
>392	13	9.05	0.32 (0.18–0.56)	<0.001	0.33 (0.18–0.58)	<0.001
Intake duration (years)						
<1	40	26.70	0.94 (0.66–1.35)	0.744	1.00 (0.70–1.44)	0.996
1.00–1.49	16	24.46	0.86 (0.51–1.46)	0.578	0.89 (0.53–1.51)	0.670
≥1.50	9	4.93	0.17 (0.09–0.34)	<0.001	0.17 (0.09–0.34)	<0.001

*DM, diabetes mellitus; HCV, hepatitis C virus; DPP-4i(–), non-dipeptidyl peptidase 4 inhibitor cohort; DPP-4i(+), dipeptidyl peptidase 4 inhibitor cohort; DDD, defined daily dose. Values were expressed as crude hazard ratios with 95% confidence intervals (CIs). After adjusting for age, sex, insurance range, comorbidities, other antidiabetic medications, anti-HCV agents, and duration of diabetes, values were expressed as adjusted hazard ratios with 95% CIs. Hazard ratios and 95% CIs with the DPP-4i(–) group as the reference group for the risk of HCC were calculated for the DPP-4i(+) group, each tertile of cumulative DDD in the DPP-4i(+) group, and each tertile of intake duration in the DPP-4i(+) group*.

## Discussion

In this study, we evaluated the risk of HCC in adult patients with type 2 DM and chronic HCV infection who did and did not receive DPP-4 inhibitor treatment from 2009 to 2013. The results showed that the use of DPP-4 inhibitors was associated with a decreased risk of HCC in these patients compared to those who did not receive DPP-4 inhibitor treatment. Furthermore, the findings revealed that a cumulative defined daily dose of DPP-4 inhibitors of more than 392 and an intake duration of more than 1.49 years were also significantly associated with a decreased risk of HCC. Our results suggested that the effect of DPP-4 inhibitors on the risk of HCC in adult patients with type 2 DM and chronic HCV infection may be related to the use, dosage and therapeutic duration of DPP-4 inhibitor treatment. The most important finding of this study is that the use of DPP-4 inhibitors was associated with a lower risk of HCC in patients with type 2 DM and chronic HCV infection. In addition, the patients who received DPP-4 inhibitors had a higher HCC-free rate.

Dipeptidyl peptidase 4 is a 110 kDa membrane-associated peptidase/cell surface glycoprotein. Dipeptidyl peptidase 4, also known as CD26 (14), is widely expressed in most cell types including lymphocytes, fibroblasts, epithelial, and endothelial cells (15–17). Dipeptidyl peptidase 4 is also a soluble protein circulating in plasma (18). High expression levels of CD26 in tumor specimens from patients with colorectal cancer have been significantly correlated with poorly differentiated tumors, late TNM stage, and the presence of metastasis (19). A high CD26 expression level has also been shown to be a predictor of poor outcomes after resection of colorectal cancer (19). In addition, Kawaguchi et al. reported that CD26 mRNA expression levels were significantly higher in tumor tissues from patients with HCC undergoing surgical resection compared to surrounding non-cancerous lesions (20). They also reported that HCC patients with a high CD26 mRNA expression had significantly larger tumors than those with a low CD26 mRNA expression (20). Moreover, Nishina et al. reported that the expression of CD26 in HCC specimens was associated with lower tumor immunity, a more advanced stage, and worse prognosis (21). Even though treating HCC cell lines *in vitro* with DPP-4 inhibitors failed to suppress cellular proliferation in these studies (20, 21), DPP-4 inhibitor treatment was shown to suppress HCC growth via the infiltration of lymphocytes into xenograft tumors or HCC *in vivo* (mice). Moreover, DPP-4 inhibitors were shown to prevent the biologically active form of the chemokine CXCL10 from being truncated by DPP-4, and CXCL10 then enhanced NK and T cell chemotaxis toward HCC cells, resulting in an antitumor effect (21). Taken together, DPP-4 inhibitors may clinically inhibit the progression and development of HCC in humans.

CXCL10 is a potent chemoattractant produced in the liver by hepatocytes and liver-infiltrating lymphocytes during HCV infection, and it can induce the migration of T-cells and NK-cells to inflamed organs to perform antiviral functions (13). Elevated concentrations of total CXCL10 have been associated with chronic HCV infection in humans (22, 23). CXCL10 binds to receptor CXCR3 on T-cells and NK-cells, resulting in the activation and migration of effector lymphocytes to the liver (13). The DPP-4-induced truncation of CXCL10 results in a form that maintains CXCR3 binding ability but does not induce signaling. Subsequently, it acts as a dominant negative modulator of the biologically active effects of the untruncated form of CXCL10 (24). Increased expressions of DPP-4 in serum and the liver have been reported in patients with HCV-related liver disease (25), and a higher concentration of truncated CXCL10 has been associated with failure to spontaneously clear acute HCV infection (13). Taken together, patients with chronic HCV infection have elevated levels of CXCL10, although this is predominantly the truncated form due to increased levels of DPP-4 caused by HCV infection, which in turns increases the chronicity of HCV infection. These findings suggest that the therapeutic inhibition of DPP-4 by DPP-4 inhibitors may prevent the truncation of CXCL10, and may be associated with decreasing the chronicity of HCV infection. Therefore, DPP-4 inhibitors may prevent the development of HCC by decreasing the chronicity of HCV infection. Yanai et al. reported that a diabetic patient with chronic HCV infection had significantly reduced HCV replication after the use of the DPP-4 inhibitor sitagliptin (26). Truncated CXCL10 has also been positively correlated with HCV-RNA and DPP-4 activity in patients with acute HCV infection (13). The use of DPP-4 inhibitors may decrease the viral load of HCV in patients with type 2 DM and chronic HCV infection. A previous study reported that serum HCV RNA titer may be an independent risk factor for the development of HCC (27). Therefore, the use of DPP-4 inhibitors may decrease the risk of HCC in patients with type 2 DM and chronic HCV infection by decreasing the viral load of HCV. Our findings are consistent with the previous studies. There are studies also shown the use of metformin in DM patients is significantly associated with reduced risk and all-cause mortality of HCC (28). In human primary hepatocytes, metformin treatment inhibited M inhibited mammalian target of rapamycin (mTOR) and phosphatase and tensin homolog (PTEN), but up-regulated p62, light chain 3B II (LC3BII) and Caspase 3. Simvastatin and metformin inhibited cell growth and HCV infection *in vitro*. In human hepatocytes, metformin increased cell-death markers. These findings suggest that Metformin together with simvastatin treatment could be useful in therapeutic prevention of HCV-related HCC (29).

The present study has some additional strengths. First, the findings can be readily generalized to the whole population because the NHI database covers 99% of the Taiwan's population. Second, the cohort was matched according to the demographic and medical characteristics of the DPP-4 inhibitor and non-DPP-4 inhibitor groups are well-matched by age, insurance range, comorbidities including HBV infection and liver cirrhosis, the use of antidiabetic medications except for DPP-4 inhibitors, the use of anti-HCV agents, and the duration of diabetes to avoid bias. Lastly, this is the first study on the association of DPP-4 inhibitor on HCC in chronic HCV infection aside from the previous animal study (21).

There are several limitations to this study. First, this was a retrospective cohort study. Although the medical records provided accurate information on prescriptions, we could not evaluate the patients' adherence to the medications. This may have resulted in underestimation of drug efficacy, especially with regards to cumulative defined daily dose and intake duration of the drug. Second, information on factors such as smoking, alcohol consumption, body weight, and body mass index which may have affected the risk of HCC was not available in the NHIRD. Third, potentially hepatotoxic medications (such as lipid-lowering agents and antifungal drugs), non-alcoholic fatty liver disease, and non-alcoholic hepatitis were not taken into consideration in this study. Finally, we considered anti-HCV therapy, but not the biochemical data of HCV parameters after anti-HCV therapy. However, few patients received anti-HCV therapy in either cohort, so the status post anti-HCV therapy may have had little influence on the risk of HCC. Further prospective studies may be needed to confirm our findings.

In conclusion, the use of DPP-4 inhibitors was associated with a lower risk of HCC in the patients with type 2 DM and chronic HCV infection in this study. The patients who used DPP-4 inhibitors had a higher HCC-free rate. This implies that DPP-4 inhibitors may prevent the development of HCC in patients with type 2 DM and chronic HCV infection. Physicians may consider prescribing DPP-4 inhibitors as second-line therapy after metformin for patients with type 2 DM and chronic HCV infection.

## Data Availability Statement

The original contributions presented in the study are included in the article/supplementary material, further inquiries can be directed to the corresponding author/s.

## Ethics Statement

The studies involving human participants were reviewed and approved by Hsueh-Wei Yen. Department of Internal Medicine, Kaohsiung Medical University Hospital, Kaohsiung Medical University. Written informed consent for participation was not required for this study in accordance with the national legislation and the institutional requirements.

## Author Contributions

M-YL: designed the study and critically revised the paper. W-HH: wrote the manuscript, analyzed, and interpreted the data. S-PS, H-LL, C-WT, and H-CL: collected the data. W-LW: analyzed the data. All authors have read and agreed to the published version of the manuscript.

## Conflict of Interest

The authors declare that the research was conducted in the absence of any commercial or financial relationships that could be construed as a potential conflict of interest.

## Publisher's Note

All claims expressed in this article are solely those of the authors and do not necessarily represent those of their affiliated organizations, or those of the publisher, the editors and the reviewers. Any product that may be evaluated in this article, or claim that may be made by its manufacturer, is not guaranteed or endorsed by the publisher.
